# Emerging roles of mitochondria in the evolution, biogenesis, and function of peroxisomes

**DOI:** 10.3389/fphys.2013.00268

**Published:** 2013-09-26

**Authors:** Abhishek Mohanty, Heidi M. McBride

**Affiliations:** Department of Neurology and Neurosurgery, Montreal Neurological Institute, McGill UniversityMontreal, QC, Canada

**Keywords:** mitochondria, peroxisome, vesicle transport, biogenesis, evolution, contact site

## Abstract

In the last century peroxisomes were thought to have an endosymbiotic origin. Along with mitochondria and chloroplasts, peroxisomes primarily regulate their numbers through the growth and division of pre-existing organelles, and they house specific machinery for protein import. These features were considered unique to endosymbiotic organelles, prompting the idea that peroxisomes were key cellular elements that helped facilitate the evolution of multicellular organisms. The functional similarities to mitochondria within mammalian systems expanded these ideas, as both organelles scavenge peroxide and reactive oxygen species, both organelles oxidize fatty acids, and at least in higher eukaryotes, the biogenesis of both organelles is controlled by common nuclear transcription factors of the PPAR family. Over the last decade it has been demonstrated that the fission machinery of both organelles is also shared, and that both organelles act as critical signaling platforms for innate immunity and other pathways. Taken together it is clear that the mitochondria and peroxisomes are functionally coupled, regulating cellular metabolism and signaling through a number of common mechanisms. However, recent work has focused primarily on the role of the ER in the biogenesis of peroxisomes, potentially overshadowing the critical importance of the mitochondria as a functional partner. In this review, we explore the mechanisms of functional coupling of the peroxisomes to the mitochondria/ER networks, providing some new perspectives on the potential contribution of the mitochondria to peroxisomal biogenesis.

## The urgency for a better understanding of peroxisomal function

Over the past decade we have learned a great deal about peroxisomal biogenesis and function, much of this using the genetic power of model organisms like yeast (Dimitrov et al., 2013; Tabak et al., 2013). However, peroxisomes are of critical importance to cellular homeostasis in mammalian systems, playing very specific and complex biochemical roles from myelination to the generation of bile (Wanders, 2013). Therefore, beyond their familiar and essential roles in beta-oxidation and the control of reactive oxygen species, peroxisomes contribute a host of specialized functions in mammalian systems. The devastating genetic diseases highlight this fact, with survival among some patients with errors in peroxisomal biogenesis between a few hours to a few years (Waterham and Ebberink, 2012). Peroxisomal genetic disorders were first defined in patients carrying mutations in the peroxisomal biogenesis/import machinery, leading to Zellweger syndrome. In these patients the primary effects is in neuronal survival, lack of myelination, and systemic muscle defects (Powers and Moser, 1998). When peroxisomes fail, there are also indirect effects on mitochondria, whose dysfunction amplifies the cellular damage (Baes et al., 1997; Baumgart et al., 2001; McGuinness et al., 2003; Dirkx et al., 2005). Exactly why mitochondria are so critically affected is unclear. However, the contribution of peroxisomal dysfunction to more common diseases like neurodegeneration, cardiovascular disease, cancer or immune disorders is only beginning to be appreciated (Fransen et al., 2013). Given the tight connection between mitochondria and peroxisomes, and the growing interest in the role of mitochondria in these diseases, it is of urgent importance that investigators examine the potential contribution of peroxisomal failure within these common human diseases. In this review we will reconsider the function and biogenesis of the peroxisomes in light of three emerging themes. First we will address their evolutionary origin, second we examine the current thinking of how peroxisomes are born in mammalian cells, and third, we focus on the functional contacts between mature peroxisomes, mitochondria, and ER in biochemical and signaling pathways. In all of these themes a common pattern emerges, where the peroxisomes have an obligate partnership with the mitochondria and the endoplasmic reticulum. We hope that a fresh look at the peroxisomes may help encourage researchers to look beyond the paradigms established from specialized, single cell experimental models and more carefully consider peroxisomal dysfunction in the etiology of complex disease pathologies.

## The evolutionary links between mitochondria, ER, and peroxisomes

The evolutionary origin of peroxisomes may provide clues to help us understand the mechanisms of peroxisomal biogenesis that occur in cells today. Opinions on this subject have changed over the years, from a purely endosymbiotic origin (De Duve, 1969), to the current evidence that peroxisomes are derived from the ER (Dimitrov et al., 2013). A bioinformatic analysis of the phylogeny of a number of peroxisomal proteins concluded that peroxisomal proteins fall into two major categories, prokaryotic and eukaryotic (Gabaldon et al., 2006). Peroxisomal proteins of eukaryotic origin (58% in yeast, 39% in rat) were primarily involved in peroxisomal biogenesis. Peroxisomal proteins with bacterial or archeabacterial ancestry included about 13–18% of the peroxisomal proteins. A large proportion of proteins were difficult to assign (~25%), but these all had some homologies with prokaryotic proteins, although trees could not be constructed to distinguish bacterial or archeal origin. Of the assigned and unassigned proteins within the second category, all were functional enzymes. This suggests either that the perixosomes have evolved as endosymbionts, or that these enzymes evolved from mitochondrial proteins sometime after the last common ancestor. Since many of these enzymes remain dually targeted to both organelles, peroxisomal biologists generally suggest that these enzymes were most likely retargeted to peroxisomes from mitochondria (Gabaldon et al., 2006; Tabak et al., 2006). This would indicate that the peroxisome emerged as functionally specialized mitochondria. Since we now know that mitochondria are able to sort specific proteins into vesicular carriers, we can begin to imagine how functionally distinct mitochondria may have taken shape. These peroxisomal precursors would have housed enzymes responsible for breaking down a unique subclass of fatty acids, incorporated specialized enzymes regulating redox pathways, and other biochemical pathways like plasmalogen synthesis. Eventually, peroxisomes would have adapted protein import mechanisms, and new signal sequences could direct precursors directly to peroxisomes. Although the genetic expansion of the peroxisomal proteome provided a great deal of independence from the mitochondria, peroxisomes have retained the same mitochondrial machinery for their division, a central aspect of peroxisomal biogenesis (Schrader et al., 2012).

If the functions of the peroxisome are largely variants of those in mitochondria, why do they emerge from the endoplasmic reticulum? This should not be particularly surprising since the ER also provides the mitochondria with the bulk of it's lipid mass, and the ER and mitochondria are functionally and physically coupled in many ways (de Brito and Scorrano, 2010; Rowland and Voeltz, 2012). Therefore, the relationship of the peroxisome to the ER may also reflect an evolutionarily conserved variation on the mechanisms of mitochondria/ER coupling. In the phylogenic analysis of the peroxisomal proteins descended from a eukaryotic lineage, there was a clear relationship between the peroxisomal import machinery and the components of the endoplasmic reticulum associated degradation, or ERAD pathway (Gabaldon et al., 2006; Schluter et al., 2006; Schliebs et al., 2010). For peroxisomal import, the receptor Pex5 binds to cytosolic precursors to deliver them to the peroxisome in a cycle that involves ubiquitination and deubiquitination of Pex5 for the release of the substrate (for review see Schliebs et al., 2010). This appears to be analogous to the use of ubiquitin in the tagging and export of unfolded proteins within the ER, which are ultimately delivered to the proteasome. Indeed 5 of the 6 conserved Pex genes show homology with components of the ERAD machinery. Pex1 and Pex6 are homologous to Cdc48 and p97 [which are themselves of bacterial origin (Iyer et al., 2004)], whereby p97 is a AAA+ ATPase. Pex2 and Pex10 are similar to the ubiquitin E3 ligase Hrd1 enzyme that tags unfolded ER proteins. Hrd1 has a binding partner Hrd3, which shows homology to Pex5, and Pex4 resembles a ubiquitin E2 ligase. Therefore, the authors concluded that the biogenesis pathway of the peroxisomes evolved from the ER (Gabaldon et al., 2006; Schluter et al., 2006; Schliebs et al., 2010). The difference is, of course that the peroxisome system would deliver rather than extract proteins. However, the only ubiquitinated cargo in peroxisomal import is actually the receptor Pex5, which is ubiquitinated in order to be extracted and recycled, following the release of the Pex5-bound import substrates in the peroxisome. In this way, the Pex1/Pex6 complex is extracting Pex5, just as the ERAD machinery extracts ER proteins (Tabak et al., 2013).

Although it has been concluded that the similarly to the Cdc48/p97 infers an ER origin of the peroxisomal import machinery, it is important to note that this system has recently been demonstrated to have a clear role at the mitochondrial membrane as well (Heo et al., 2010; Tanaka et al., 2010; Chan et al., 2011; Xu et al., 2011; Esaki and Ogura, 2012). In this case, targeted substrates are ubiquitinated by E3 ligases such as Parkin, a protein that is mutated in familial cases of Parkinson's disease. p97 is required for the retrotranslocation of these tagged proteins, which are then targeted to the proteasome for degradation. Therefore, we would argue that this homology does not exclusively implicate the ER as the membrane of origin for the peroxisomal import machinery, and equally supports a mitochondrial origin.

## Beyond evolution: cell biology of peroxisomal biogenesis today

A number of studies using yeast as a model organism have unequivocally demonstrated that peroxisomes can be formed *de novo* from the endoplasmic reticulum (Dimitrov et al., 2013; Tabak et al., 2013). This information has effectively shelved the notion that peroxisomes evolved as endosymbionts. Unlike mammalian cells, yeast govern their peroxisomal numbers depending on the carbon source, for example in the presence of oleic acid (*Saccharomyces cerevisiae* or *Yarrow lipolytica*) (Trotter, 2001) or methanol (*Hansenula polymorpha* and *Pichia pastoris)* (Yurimoto et al., 2011). Since yeast mitochondria do not perform beta-oxidation, peroxisomes rapidly arise from the ER in order to catabolize these fats, or to metabolize methanol. In this way, fungi are highly specialized organisms where peroxisomal function has diverged between evolutionary lineages. On the other hand, the linkages to the mitochondria are much more obvious in multicellular organisms. For example, the transcriptional regulation of mitochondria and peroxisomal biogenesis is not coupled in yeast as it is in mammals (Issemann and Green, 1990; Mandard et al., 2004; Scarpulla et al., 2012). In addition, the shared roles of peroxisomes and mitochondria as signaling platforms (Dixit et al., 2010; Tait and Green, 2012) may not occur in yeast, and most obviously, the metabolic functions of peroxisomes have diverged significantly throughout evolution (Islinger et al., 2010; Pieuchot and Jedd, 2012; Wanders, 2013). Therefore, fungal lineages may have lost some of the linkages between the mitochondria and peroxisomes, instead developing closer ties to the ER. We consider that there is likely a great deal of plasticity in the evolution of peroxisomes, depending on the specific functional role they play across diverse species. Given this divergence, we suggest that there may not be unified theory for peroxisomal biogenesis across species, where, for example, significant differences are likely to exist between yeast and mammalian mechanisms.

The most compelling evidence to demonstrate the contribution of the ER to peroxisomal biogenesis is the emergence of Pex-containing vesicles from the endoplasmic reticulum in yeast and mammals. A number of different experimental paradigms and model systems have proven this point. First, fluorescently tagged, membrane anchored Pex proteins, notably Pex3 and Pex16, have been observed emerging from the ER in conditions where peroxisomes are either induced by growth conditions or in pulse-chase type of rescue experiments (Titorenko and Rachubinski, 1998; Hoepfner et al., 2005; Kragt et al., 2005; Tam et al., 2005; Kim et al., 2006; Motley and Hettema, 2007). Second, cell free budding assays from isolated ER have established some of the machinery required to bud Pex-containing vesicles in yeast *Saccharomyces cerevisiae* (Lam et al., 2010). In this case, the authors showed both Pex3p and Pex15p emerging within vesicles in a manner that depended on ATP and Pex19p, but not Sar1, a GTPase essential for anterograde COPII budding events. The authors demonstrated a requirement for additional cytosolic factors that are yet to be identified. Using a semi-permeable cell system in *Pichia Pastoris*, the authors also demonstrated a Pex19 dependent, COPII-independent mechanism to generate Pex11-containing vesicles from the ER (Agrawal et al., 2011). These vesicles were generated even in the absence of Pex3, an essential component for peroxisome biogenesis. Similar data has shown an ER origin for mammalian peroxisomes. Using human fibroblasts lacking core proteins of the peroxisomal import machinery like Pex16 or Pex3, the reintroduction of GFP-tagged Pex16 or Pex3 can rescue the generation of new organelles from their ER localization (Kim et al., 2006; Toro et al., 2009; Yonekawa et al., 2011). This pathway was also shown to depend upon the ER budding factor Sec16b (Yonekawa et al., 2011). Although many peroxisomal proteins target the mitochondria in the absence of peroxisomes (see next section for further discussion), these data clearly establish the ER as a primary source of membrane in the generation of new peroxisomes.

How can you generate a mature peroxisome from an ER derived vesicle? Historically it has been assumed that the early peroxisomes would be import competent, and from there could mature through the targeting and import of all the required functional enzymes. This maturation model did not require any fusion events, instead all new peroxisomes would be formed from the growth and division of existing peroxisomes. However, now it is clear that small, vesicular carriers bud from the ER, which are termed “pre-peroxisomes.” These vesicles must fuse with other pre-peroxisomes, or with more mature peroxisomes, to generate a larger, functional organelle (Boukh-Viner et al., 2005; van der Zand et al., 2012). Many studies have proven that mature peroxisomes do not fuse, both in mammalian or yeast cells (Motley and Hettema, 2007; Huybrechts et al., 2009; Bonekamp et al., 2012), raising the important question of specificity and regulation of fusion among/between pre-peroxisomes. Previous work in yeast *Yarrow lipolytica* demonstrated peroxisomal fusion *in vitro*, and it was suggested then that fusion was limited to an “early” pool of peroxisomes that would then mature into fully functional organelles (Titorenko et al., 2000; Boukh-Viner et al., 2005). In *Yarrow lipolytica*, peroxisomal fusion was dependent upon the import factors Pex1 and Pex6, of the Cdc48/p97 family (Titorenko et al., 2000; Boukh-Viner et al., 2005). Although p97 is a AAA+ATPase that functions in the ERAD pathway, p97/Cdc48 have also been shown to have an established role in ER and golgi membrane fusion (Latterich et al., 1995; Hetzer et al., 2001; Uchiyama et al., 2006; Totsukawa et al., 2013).

Another important question is how the fusion of two pre-peroxisomes would lead to a functional peroxisome. Recent work has answered this conundrum by revealing that the peroxisomal import machinery is sorted into two populations within the ER. They observed two distinct populations of vesicles budding from the ER, one containing the RING complex of the import machinery, and the second carrying the docking complex (van der Zand et al., 2012). Heterotypic fusion between these two distinct populations of pre-peroxisomes would then generate a functional import machine, and by definition, a functional peroxisome. This explains why peroxisomal import cannot occur into the ER, since the machinery remains segregated. The mechanisms for this segregation are not yet known. Consistent with previous work, the authors could not observe any fusion events between pre-peroxisomes and mature peroxisomes, or between mature peroxisomes, indicating a highly selective mechanism for pre-peroxisomal fusion (van der Zand et al., 2012).

## Is there a potential role for mitochondrial derived vesicles (MDVs) in peroxisomal biogenesis?

Since the emergence of peroxisomes from the ER is so clearly demonstrated, is there any role for the mitochondria in the biogenesis of peroxisomes beyond their evolutionary links? Certainly our understanding of the flexibility of mitochondria has rapidly increased, and we appreciate how they fuse and divide in order to dynamically position themselves both functionally and spatially within cells. Our lab has also defined two distinct vesicular transport routes from the mitochondria (Neuspiel et al., 2008; Braschi et al., 2010; Soubannier et al., 2012a,b). Initially we described a route between the mitochondria and the peroxisome (Neuspiel et al., 2008), a pathway we also showed was dependent upon the retromer complex Vps35, Vps26 and Vps29 (Braschi et al., 2010). More recently we characterized another pathway between the mitochondria and the late endosome/multivesicular body. This latter pathway selectively targets oxidized or damaged protein and lipid (Soubannier et al., 2012b), removing them from the mitochondrial reticulum for degradation in the lysosome (Soubannier et al., 2012a). These two pathways open up new insights into how the mitochondria may deliver their contents to other cellular organelles.

What is the function of the vesicle transport route from the mitochondria to peroxisomes? So far only one cargo was identified in these vesicles, a membrane anchored protein called MAPL (mitochondrial anchored protein ligase, also called MUL1, GIDE, or HADES) (Neuspiel et al., 2008; Braschi et al., 2009). The carboxy-terminal of MAPL is exposed to the cytosol and contains a RING finger domain with strong SUMO E3 ligase activity. Overexpression of MAPL drove massive mitochondrial fragmentation through the SUMOylation and activation of the mitochondrial fission GTPase Drp1 (Neuspiel et al., 2008). Mitochondrial vesicles carrying MAPL fused with a subset of peroxisomes, only about 10% of the total (Neuspiel et al., 2008; Braschi et al., 2009). These peroxisomes were able to import the transfected CFP-SKL marker, indicating that they had functional import machinery. Although we have not yet determined the function of this vesicular transport route, we offer three potential functions here. First, MDVs may contribute to peroxisomal biogenesis, fusing with the early, preperoxisomal population. Second, MDVs may carry metabolites and target a functionally distinct subset of peroxisomes, and third, MDVs may shuttle proteins that are not competent for peroxisomal import.

Given the evidence for a pre-peroxisomal population in cells, it is plausible that MAPL is targeted to this fusogenic population of peroxisomes and provides a mitochondrial component to the maturing peroxisomes. In evolutionary terms, a phylogenic analysis of MAPL indicates that it is of bacterial ancestry, with at least 5 prokaryotic domain structures (Andrade-Navarro et al., 2009). Therefore, this vesicle transport pathway may have played a role in the earliest segregation of specialized mitochondrial function. This possibility has not been previously considered due to the obvious assumption that the mitochondria were not competent to segregate cargo and bud vesicles. This assumption is fundamentally wrong. Our ongoing studies continue to characterize various classes of cargoes that are enriched in mitochondrial derived vesicles. For example, using an *in vitro* reconstitution system we demonstrated that the identity of the cargo within MDVs destined for the lysosome depends greatly on the nature of the insult (Soubannier et al., 2012b). We have a great deal of work ahead to identify the mechanisms and regulation of mitochondrial vesicle transport, but it is clearly a process that exists in steady-state conditions, suggesting a fundamental role for these vesicles in cellular homeostasis.

**Figure 1:**
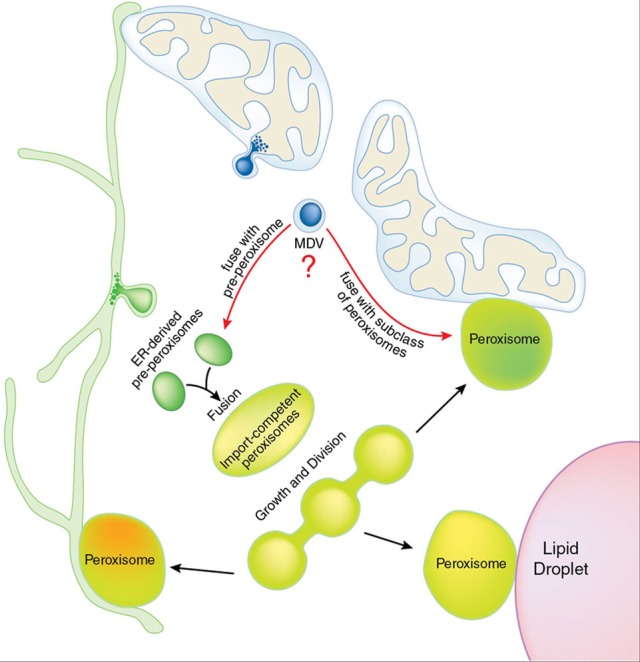
**Potential contribution of MDVs to peroxisomal biogenesis and functional specialization**. Pre-peroxisomes bud from the endoplasmic reticulum (ER, in green), carrying subcomplexes of the import machinery. Fusion between ER-derived pre-peroxisomes results in an import competent peroxisome that continues to grow and mature. Elongation and division of peroxisomes occurs throughout the life of peroxisomes, in response to cellular conditions. Mitochondria are shown (blue) with a mitochondrial derived vesicle (MDV) emerging, containing both inner and outer mitochondrial membranes. Both membranes were observed in immuno-electron microscopy analysis of MAPL-positive vesicles, indicating that double-membrane bound vesicles target the peroxisome (Neuspiel et al., 2008). Since MAPL containing MDVs fuse with only a sub-population of peroxisomes, we envision two possible fates (indicated by red arrows). First, MDVs may fuse with an early pre-peroxisomal pool. MAPL was seen to fuse with peroxisomes containing CFP-SKL (Neuspiel et al., 2008; Braschi et al., 2010), indicating that MDVs fuse with an import-competent class of organelles. From this we predict that MDV fusion would occur downstream of ER-derived pre-peroxisomal fusion. Alternatively, MDVs could fuse with a functionally distinct subclass of peroxisomes, which are illustrated by different shading within mature peroxisomes. A number of direct contact sites are shown between organelles, which have been characterized in many cellular conditions (see text for details).

If mitochondrial vesicles play a role in peroxisomal biogenesis, why don't we observe peroxisomal membrane proteins targeting the mitochondria? Indeed, in mammalian cells, many peroxisomal proteins do default to the mitochondria when peroxisomes are absent. For example, Pex3, Pex14, Pex12, PMP70 and ALDP/ABCD1 were shown to target mitochondria in fibroblasts of patient cells with mutations in Pex3, Pex16 or Pex19 (Sacksteder et al., 2000; South et al., 2000; Kim et al., 2006; Toro et al., 2009). This has been generally discounted as an artifactual missorting, again, likely since it had been assumed that there was “no way out” for these mistargeted proteins. In contrast, yeast biologists did not consider sorting to the ER in peroxisome-deficient cells to be an artifact; rather it defined the ER as the site of peroxisomal biogenesis. In light of a vesicular transport route from the mitochondria to the peroxisomes, we should reconsider these older studies and realize that this supports the concept that mitochondria may contribute to the initiation of new peroxisomes in mammalian cells. The most consistent explanation is that both ER and mitochondrial derived vesicles could contribute to new peroxisomes (Figure 1). It is possible, for example that some pre-peroxisomes derived from the ER could fuse with pre-peroxisomes derived from the mitochondria.

The plasticity of these pathways is illustrated by comparing rescue experiments in different systems. For example, the reintroduction of Pex3-GFP into a mammalian cell line lacking Pex3 led to the generation of new peroxisomes, which emerged via ER-localized Pex3-GFP, not the mitochondria (Toro et al., 2009). On the other hand, when the Erdmann group ectopically targeted Pex3 to the mitochondria within Pex3-null yeast strains (Rucktaschel et al., 2010), peroxisomes were successfully regenerated from the mitochondria. Therefore, although an emerging pathway in peroxisomal cell biology, mitochondrial vesicles should be explored as potential contributors to the biogenesis of peroxisomes, particularly in mammalian cells. There may be significant variability in the relative contribution of ER and mitochondrial derived vesicles to peroxisomal biogenesis, which would likely be linked to the functional diversity of the organelles in different tissues and organisms.

It is also possible that mitochondrial derived vesicles could carry cargo to functionally distinct peroxisomes, rather than playing a role in their biogenesis. There has been evidence that peroxisomes can be functionally differentiated within single cells, having distinct densities, import competencies, and protein composition (Schrader et al., 1994; Fahimi et al., 1996; Volkl et al., 1999; Islinger et al., 2012; Costa et al., 2013). However, it is not known how this might be achieved. Given the functional coupling of the mitochondria and the peroxisomes—particularly in mammalian cells—a direct vesicular transport route may allow the rapid shuttling of metabolites or proteins with very tight spatial and temporal regulation. A vesicular transport route could selectively target “active” peroxisomes and deliver metabolites or enzymes selectively to these organelles (Figure 1). Vesicles may also provide protection from potentially toxic, hydrophobic, or more complex mitochondrial cargoes. These could include heme, lipids like mitochondrial-generated PE or cardiolipin (Wriessnegger et al., 2007), or metabolic intermediates. It has been largely concluded that catalase is imported into peroxisomes in the heme-loaded form, indicating that heme would be an unlikely cargo for MDV transport. However, earlier work by Lazarow and DeDuve used radiolabelled pulse-chase experiments in rat liver elegantly demonstrated that heme loading of catalase occurred only after import (Lazarow and de Duve, 1973). Again, it cannot yet be excluded that some heme could be transported into peroxisomes from the mitochondria in vesicular carriers.

Finally, it is also possible that the mitochondrial protein import and folding machinery is more efficient than peroxisomes, leading some common enzymes to be shipped to peroxisomes only after their rapid assembly in the mitochondria. MAPL, with it's two transmembrane domains may fall into this category. Dually targeted proteins could potentially utilize both transport routes, depending on the conditions.

There are a number of challenges remaining to identify the molecular machinery that regulates this vesicular transport route, to understand which types of cargoes are segregated into the vesicles, and how these vesicles select and fuse with a subset of peroxisomes. Answers will likely come from large scale screening efforts and the development of cell-free assay systems. Such a screen of 4000 viable deletions in yeast provided compelling evidence for a direct link between the mitochondria and peroxisomes. This genome-wide scan of factors affecting peroxisomal biogenesis in yeast identified only 4 ER proteins, but 41 mitochondrial proteins whose loss affected peroxisomal numbers, shape, or function (Saleem et al., 2010). This can be compared to the loss of 46 nuclear-targeted proteins that similarly affected the peroxisomes. Surprisingly, none of the 4 ER proteins are known to function in vesicle formation, instead were mapped to fatty acid synthesis, farnysylation, lipid modifications and signal transduction (Saleem et al., 2010). Clearly many ER proteins, including many of those required for ER transport are essential, and therefore would not be seen in this screen of non-essential genes. On the other hand, the mitochondrial genes spanned multiple functions, from mitochondrial translation to respiration and mtDNA distribution. The robust effects on peroxisomal function upon the loss of so many mitochondrial genes should reinforce our efforts to consider the dynamic interplay between peroxisomes and mitochondria in all organisms. Also notable in this screen was the fact that the loss of 9 vacuolar proteins also led to a reduction in peroixomal numbers and content (Saleem et al., 2010). These proteins included fusion factors like the SNAREs Vam3 and Nyv1, and the Rab GTPase, Ypt7. Future work will be required to understand the functions of these genes in peroxisomal behavior.

## Peroxisome and mitochondrial dynamics: the implications of a shared mechanism

One of the most striking parallels between the mitochondria and peroxisomes is the conservation of the fission machinery in diverse organisms from yeast to plants. Deletion of the dynamin-related protein Drp1 [also called Dlp1 (dynamin-like protein) and Dnm1 (in yeast)] led to elongated peroxisomes and mitochondria (Koch et al., 2003; Kuravi et al., 2006; Kobayashi et al., 2007; Motley et al., 2008). The recruitment of this cytosolic GTPase requires its single-membrane spanning receptors Mff and Fis1, which are dually imported into both organelles (Koch et al., 2005; Delille and Schrader, 2008; Motley et al., 2008). Other mitochondrial fission factors, including GDAP1 are also imported into peroxisomes in a Pex19-dependent manner (Huber et al., 2013). Interestingly, MAPL activates mitochondrial fission through the SUMOylation of Drp1 (Braschi et al., 2009). As described above, MAPL is a cargo that is transported to the peroxisomes in mitochondrial derived vesicles (MDVs) (Neuspiel et al., 2008; Braschi et al., 2010). Since MAPL was delivered only to a subpopulation of peroxisomes in HeLa cells, it suggests that MAPL-mediated activation of peroxisomal fission would be specific to either early peroxisomes, or functionally specialized organelles. Our lab continues to work on this pathway to elucidate the impact of MAPL on peroxisomal function and dynamics. Peroxisomes employ other factors, including the family of Pex11 proteins, which promote peroxisomal elongation (Koch et al., 2004; Kobayashi et al., 2007), indicating that there are also organelle specific factors regulating their division.

Having established that mitochondrial and peroxisomal fission utilize common machinery, what does this mean for the cell? On one hand, this fact further supports the idea that the mitochondria are a contributor to the ancestry of the peroxisome. But of more immediate relevance, it suggests that the mechanisms and signaling pathways that activate the fission machinery of the two organelles are coupled. In other words, when mitochondria fragment, peroxisomes should also fragment. For example, is Drp1 also stably recruited to peroxisomes during the apoptotic program, and would this contribute to the mechanisms of cell death (Frank et al., 2001; Wasiak et al., 2007)? In contrast, the inhibition of Drp1 by PKA phosphorylation during autophagy could trigger peroxisomal elongation (Gomes et al., 2011; Rambold et al., 2011). Would this affect the breakdown of fatty acids during starvation to promote gluconeogensis? Are longer peroxisomes also functionally more efficient, or resistant to degradation by pexophagy? These are important questions that will hopefully be answered soon.

Recent studies in mitochondrial fission have also highlighted a critical role for the endoplasmic reticulum in defining the site of Drp1 scission sites (Friedman et al., 2011; Murley et al., 2013). Does Drp1 recruitment somehow mark a site to tether the peroxisomes to the ER? Is the ER functionally required to mark sites of peroxisomal division? These are questions that are likely the topic of current investigation in many labs. There has been some advance in our understanding of how peroxisomal division in yeast may be regulated by signaling pathways, through the peroxisome-specific fission factor Pex11. Yeast Pex11 was shown to be phosphorylated in both *Pichia pastoris* and *Saccharomyces cerevisiae* in the presence of oleate (Knoblach and Rachubinski, 2010; Joshi et al., 2012). In *Saccharomyces* the phosphorylation was mediated by a cyclin-dependent kinase Pho85p, potentially linking peroxisomal fragmentation, and segregation during the cell cycle (Joshi et al., 2012). In *Pichia*, Pex11 phosphorylation facilitated an interaction with Fis1 in steady state (Knoblach and Rachubinski, 2010). This provides further evidence that peroxisomal dynamics are tightly regulated through signaling cascades in multiple organisms. Much more work remains to be done to fully understand the functional implications of peroxisomal length, and whether/when fission may be controlled by signaling pathways.

Another emerging aspect of peroxisomal and mitochondrial dynamics is the contribution of organelle plasticity to quality control. It is clear that functionally aberrant, or damaged mitochondria or peroxisomes must be removed in order to ensure the survival of the cell. For peroxisomes, the primary mechanism is through pexophagy, where the autophagic machinery engulfs “old” peroxisomes for degradation in the autophagosomes (Nordgren et al., 2013). This implies that there may be conserved mechanisms that target Drp1 selectively to fission sites where dysfunctional organelles will be removed. However, a role for Drp1 in pexophagy has not yet been established.

Peroxisomes also contain a number of proteases to degrade unfolded or misassembled complexes, including the LonP2 enzyme which is paralagous to the mitochondrial LonP, and degrades oxidized proteins in a similar manner (Kikuchi et al., 2004). Finally, retrotranslocation pathways called RADAR for Receptor Accumulation and Degradation in the Absence of Recycling, functions in a ubiquitin-dependant manner similar to the ERAD pathway of removal from the ER (Leon et al., 2006; Leon and Subramani, 2007). Whether errors in these pathways are a primary cause of disease is something that is becoming a very important area for future research.

A newly identified mechanism for mitochondrial quality control is also the use of vesicular carriers that selectively remove oxidized proteins and lipids from otherwise intact organelles (Soubannier et al., 2012a,b). Interestingly, peroxisomes within fungi like *Neurospora crassa* are known to segregate assembled complexes of Hex-1 protein oligomers that pinch off into specialized organelles called woronin bodies. These structures target and physically block the leakage of hyphal contents within broken fungal branches (Tenney et al., 2000; Tey et al., 2005). Mechanistically the Hex-1 oligomers have been shown to interact with peroxisomal protein import components within a subclass of peroxisomes, which stimulates import to fuel the generation of Hex-1 crystals (Liu et al., 2011). This process effectively “differentiated” this subclass of peroxisomes to function in the generation of Hex-1 crystals rather than their other functions in redox control or beta-oxidation. This segregation was effectively reconstituted in a yeast model ectopically expressing the Hex-1 protein. The generation of the Hex-1 containing woronin bodies in this system was dependent on Dnm1/Vps1 for their division (Wurtz et al., 2008). In addition, peroxisomes in the yeast *Hansenula polymorpha were shown to segregate mutant catalase aggregates through a fission-dependent process, which were targeted to the autophagosome* (Manivannan et al., 2013). This indicates that peroxisomes also have a capacity to segregate cargo for their selective removal. Whether this processes involves the generation of small vesicles (~100 nm with coat proteins, cargo enrichment mechanisms, etc.), or is done exclusively through the segregation and Drp1-dependent fission of larger, non-vesicular structures (or both) needs to be further explored. In any case, the segregation of cargo is a specific process in cell biology that requires complex mechanisms and regulation.

A decade ago the field of mitochondrial dynamics was largely considered phenomenological. However, time has proven the fundamental importance of mitochondrial shape and position in the regulation of mitochondrial function (Nunnari and Suomalainen, 2012). A similar future awaits the field of peroxisomal dynamics. At least in mammalian systems, the functional consequences of precise peroxisomal positioning and contacts within the cell, and the question of regulated division and elongation during various cellular transitions is primed for new discovery.

## The habits of a mature peroxisome

Functional peroxisomes have mechanisms for selective protein turnover (Nordgren et al., 2013), but the organelle itself is thought to remain stable within the cell for 1–4 days (Price et al., 1962; Poole et al., 1969). During this time proteins are imported, and they perform a number of major functions including the beta oxidation of very long chain fatty acids, the breakdown of peroxide, and the synthesis of specific compounds like bile acids, ether phospholipids like plasmalogen, etc (Wanders, 2013). Some of these functions, like the generation of plasmalogen, involve biochemical pathways present in anaerobic bacteria, further supporting a prokaryotic lineage for peroxisomal enzymes (Goldfine, 2010). However, the habits of peroxisomes in fungi, plants, and animals can vary widely, where entire biochemical pathways have been lost and/or expanded across the species, from fungi to plants and animals (Islinger et al., 2010). The generation of plasmalogens is one example, and the synthesis of bile is also specific to animals. For each of these pathways, the substrates and products of reactions performed within peroxisomes are acquired from, or targeted to, other cellular organelles. Historically, metabolite transport was assumed to occur by free diffusion, without requiring any specific contact sites. However, emerging cell biological studies continue to highlight the importance of direct organelle contacts between peroxisomes and the ER, mitochondria, and lipid droplets (Schrader et al., 2013). The task ahead is to determine the molecular mechanisms and regulation of these contacts, and determine whether these contacts really play an essential role in the funneling of metabolites. If so, it is conceivable that functionally distinct peroxisomes may favor contacts with just one partner organelle (i.e., mitochondria, ER, or lipid droplets), leading to a type of peroxisomal differentiation within single cells (Figure 1). Given the technical limitations in visualizing peroixisomal metabolism within living cells, we cannot yet distinguish these possibilities. As cell biologists, the concept of free diffusion is very unappealing given the kinetic disadvantages compared to regulated, targeted interorganelle transport and direct contact (Howe, 2005). Without entering into the biochemical details of peroxisomal metabolism which are elegantly described elsewhere (Wanders, 2013), we describe three examples of the functional contacts that are currently under investigation in various cell models.

Plasmalogen is an ether phospholipid generated from enzymes in both the peroxisomes and the ER (Braverman and Moser, 2012). Once it is synthesized, plasmalogen is localized with the ER, but more significantly within golgi membranes, mitochondria and the nucleus. However, the bulk of plasmalogen is secreted from cells, and used in a variety of processes including the generation of myelin (in brain), surfactant (in lung), and in the development of the lens in the eye (Gorgas et al., 2006). Many of the severe phenotypes in patients with peroxisomal deficiencies are due, in large part, to a loss in plasmalogen biosynthesis. The first three enzymes of this pathway are localized in the peroxisomes, and the last three enzymes reside in the ER. Plasmalogen synthesis begins with a fatty acid, which is likely stored in the lipid droplet. A peroxisomal surface enzyme called FAR1 converts the fatty acid into a fatty alcohol, which enters the peroxisome (Honsho et al., 2010). Given the close contact of the peroxisomes with both the lipid droplets and ER, it has been suggested that “kiss and run” events help to facilitate the transfer of substrates and products between these organelles (Schrader et al., 2013). Indeed, lipids are transported from the ER into the mitochondria through well-established contact sites called MAM, for microsomal associated microdomains (English and Voeltz, 2013). Similar sites appear to exist between the ER and mature peroxisomes (Raychaudhuri and Prinz, 2008), however, the molecular basis for these contacts is unknown.

Peroxisomes are also essential in the production of bile acid salts, which are secreted from the liver to emulsify dietary fats travelling through the gut (Ferdinandusse et al., 2009; Lefebvre et al., 2009). The two fatty acids Di- and Trihydroxycholestanoic Acid (DHCA and THCA) are produced from cholesterol in the ER, and are then transported to the peroxisomes, likely through PMP70/ABCD3 (Morita and Imanaka, 2012). Following a few rounds of beta-oxidation in peroxisomes, the resulting acetyl-CoA esters are converted to the taurine and glycine conjugates for export back into the cytosol. These bile acids are then released from the hepatocytes into the bile caniliculi, which can be stored in the gall bladder and secreted into the gut. Therefore, as in the synthesis of plasmalogens, the generation of bile acid involves the transport of metabolites between the ER, the peroxisomes, and the plasma membrane. Whether or not the peroxisomes are in contact with the plasma membrane for direct flux of the bile salts across the membrane has not been explored.

The most conserved function for peroxisomes is the beta-oxidation of very long chain fatty acids. In fungi like yeast, peroxisomes are responsible for the beta-oxidation of all fatty acids, therefore there is no obvious requirement for any mitochondrial/peroxisomal contacts in these organisms. However, yeast grown in the presence of oleic acid were shown to trigger significant direct contacts between peroxisomes, mitochondria and the lipid droplet, hinting toward the direct transfer of fatty acids through these contact sites (Pu et al., 2011). In higher eukaryotes, peroxisomes catabolize very long chain fatty acids, and transport the medium chain products and acyl-CoA moieties into the mitochondria for further oxidation (Wanders, 2013). Therefore, beta-oxidation in mammals likely involves direct contacts between the peroxisomes and mitochondria, although links to the lipid droplets are likely also implicated. Whether or not any lipids or substrates could be transported in vesicular carriers between these organelles is also unknown.

In all of these instances, as well as numerous biochemical pathways we haven't described here, there is a constant need for the peroxisomes to be in direct contact with various intracellular organelles. The primary partners are the ER and the mitochondria, although there is evidence for contacts with lipid droplets as well. Future work will continue to explore the functional importance and molecular specificity of these contacts.

## Peroxisomes and mitochondria as unique signaling platforms

As a final comment on the functional coupling between the peroxisomes and the mitochondria, we end with their important roles in intracellular signaling pathways. A well-established core function of both the mitochondria and peroxisomes is their ability to scavenge damaging reactive oxygen species or peroxides (Starkov, 2008; Bonekamp et al., 2009). ROS scavenging is important to minimize cellular damage, but the contribution of ROS to signaling pathways is of equal importance (Tschopp, 2011; Murphy, 2012; Sena and Chandel, 2012). Recent studies utilizing a peroxisomal-targeted redox probe in both mammalian and yeast cells demonstrated significant variation in peroxisomal redox state depending on the environmental conditions (Ivashchenko et al., 2011). Overall the peroxisomes and mitochondria exhibited much lower levels of oxidation than expected, given the focus on these organelles as hot beds of reactive species. The strict control over the levels of ROS in these organelles reaffirms their competence in neutralizing damage to protect the cell. As seen earlier in a number of peroxisomal mutant fibroblasts (Baumgart et al., 2001; Dirkx et al., 2005), disruption of peroxisome redox status adversely affected the mitochondrial redox state, further highlighting the functional links between the two (Ivashchenko et al., 2011). In these experiments, individual peroxisomes with very high oxidative status were eliminated through pexophagy, consistent with the concept of selective autophagy (Nordgren et al., 2013). Whether the redox status of the mitochondria and peroxisomes feeds back into changes within the ER remains unexplored.

Although the mitochondria and peroxisomes are able to minimize the accumulation of reactive species, this does not exclude a role for a highly localized and/or situation-specific use of oxidation mechanisms in signaling. Our own work investigating the molecular mechanisms that drive stress-induced mitochondrial fusion has shown that elevations in oxidized glutathione (GSSG) lead to the oligomerization and “priming” of the mitochondrial fusion GTPases Mfn1 and Mfn2 (Shutt et al., 2012). This has led us to consider a global role for increased local oxidation as a means to initiate protein modifications that may lead to their activation. A second example of this is the more established redox sensor KEAP1, which normally targets the Nrf2 transcription factor for ubiquitination by a Skp/Cul3 ubiquitin ligase complex (Itoh et al., 2010). Upon increasing levels of GSSG, new disulfide bonds are formed within KEAP1, rendering it unable to bind Nrf2, which is then targeted to the nucleus where it transcribes a host of stress response genes. KEAP1 has been localized to the mitochondrial surface, through its interaction with the mitochondrial outer membrane protein PGAM5 (Lo and Hannink, 2008), suggesting that local redox transitions at the mitochondria could effectively control Nrf2 transcriptional responses.

Perhaps the most surprising links between the mitochondria and signaling pathways came a number of years ago with the identification of the mitochondrial anti-viral signaling protein MAVS. MAVS was identified from 4 independent groups simultaneously as an essential protein for the viral-induced transcription of Nf-kB (Kawai et al., 2005; Meylan et al., 2005; Seth et al., 2005; Xu et al., 2005). Only one of the 4 groups examined this protein by microscopy and realized that it is a mitochondrial membrane anchored protein (Seth et al., 2005). MAVS contains a carboxy-terminal transmembrane domain and a cytosolic CARD domain. Upon infection, the cytosolic double-stranded viral DNA forms a complex with RIG-I, which binds the CARD domain of MAVS at the mitochondrial surface. From there, a complex series of protein interactions and oligomerization steps leads to the formation of extremely large, prion-like MAVS filaments (Hou et al., 2011; Berke et al., 2012). These filaments are even “contagious” as they can seed the formation of MAVS filaments in ectopic situations.

MAVS has also been seen to signal even earlier from the peroxisomes, again linking these two organelles as unique signaling platforms (Dixit et al., 2010). So why do these things occur on the mitochondrial or peroxisomal surface? Initially the mitochondrial localization of MAVs suggested some role in delaying apoptosis until the infected cell could secrete cytokines to alert the neighboring cells. However, this has been challenging to prove, and the localization upon non-apoptotic peroxisomes suggests something different. For example, one of the core observations during infections is the spike of ROS that occurs, and has been shown to play a critical role in the host response (Soucy-Faulkner et al., 2010). There is evidence that mitochondria, and likely peroxisomes, contribute to these ROS spikes (Sena and Chandel, 2012). As ROS levels increase on the surface of these organelles, it opens the possibility that transient disulfide switching may mechanistically activate the MAVS complexes (Xiong et al., 2011). The evolution of conserved, redox-sensitive cysteine residues within MAVS or associated proteins could help explain why these complexes target the mitochondria and peroxisomes. More recently, MAVS was shown to recruit the inflammasome to the mitochondrial surface, a process specific for certain classes of activators (Subramanian et al., 2013). So far the MAVs regulated complexes appear to be specific to innate rather than adaptive immunity. A common theme in immune activation is the requirement for ROS spikes upon infection (Tschopp, 2011). Therefore, we suspect that the reason the mitochondria and peroxisomes are commonly used as signaling platforms is due to the high local concentrations of ROS (and subsequently oxidized glutathione) that can trigger conformational changes through disulfide switching mechanisms. Future work will continue to explore these and other hypothesis.

## Concluding remarks

In this review we have highlighted a series of observations that illustrate the very tight functional, spatial, and regulatory links between the peroxisomes and the mitochondria. Evolutionary analysis coupled with the emergence of a vesicular transport route between the mitochondria and peroxisomes propels us to consider a role for mitochondria in peroxisomal biogenesis. Since ER-derived pre-peroxisomes are fusogenic (Boukh-Viner et al., 2005; van der Zand et al., 2012), and the mitochondrial cargo MAPL was seen to fuse with only a subpopulation of peroxisomes (Neuspiel et al., 2008; Braschi et al., 2010), we hypothesize that MDVs may contribute to early peroxisomal formation. Mature peroxisomes are also tightly integrated within complex biochemical cascades, funneling their substrates and products to the mitochondria, ER and sometimes lipid droplets (Schrader et al., 2013). Therefore, an alternative to a role in biogenesis is that MDVs could also selectively deliver metabolites to functional subclasses of peroxisomes within a cell. An analysis of the extensive metabolite flux required to flow between these organelles helps to fuel our speculation about functional specialization among peroxisomes. Clearly there is a great deal of work to do in order to distinguish these possibilities. Finally we proposed a general hypothesis where local oxidation may be used to activate cellular signaling pathways, which may explain why the mitochondria and peroxisomes work together as unique signaling platforms.

The critical importance of peroxisomes in physiology is chronically underappreciated within the wider scientific community. Along with their established links to the ER, we hope that increasing awareness of the obligate coupling of the peroxisomes to the mitochondria will encourage researchers to more carefully consider the contribution of peroxisomal dysfunction to disease progression. For example, a great deal of attention is currently being paid to the role of mitochondria in neurodegeneration, cancer and immunology, yet the impact of mitochondrial dysfunction on peroxisomes is virtually unexplored in these disease pathologies. There is a great deal of work to be done before we will fully understand the role of peroxisomal dysfunction in human disease. A first step will require a better characterization of the molecular mechanisms that regulate the behavior and biochemistry of peroxisomes as a dynamic and tightly integrated organelle.

### Conflict of interest statement

The authors declare that the research was conducted in the absence of any commercial or financial relationships that could be construed as a potential conflict of interest.
